# Apparent diffusion coefficient measurements of bone marrow infiltration patterns in multiple myeloma for the assessment of tumor burden – a feasibility study

**DOI:** 10.2478/raon-2023-0048

**Published:** 2023-11-30

**Authors:** Xing Xiong, Yuzhu Ma, Yao Dai, Chunhong Hu, Yu Zhang

**Affiliations:** Department of Radiology, The First Affiliated Hospital of Soochow University, Suzhou, Jiangsu, China; Department of Radiology, Dushu Lake Hospital Affiliated to Soochow University, Suzhou, Jiangsu, China

**Keywords:** multiple myeloma, bone marrow, infiltration pattern, tumor burden, apparent diffusion coefficient

## Abstract

**Background:**

The purpose of our study was to explore and compare the tumor burden of different bone marrow infiltration patterns and evaluate the feasibility of apparent diffusion coefficient (ADC) value to identify patterns in multiple myeloma (MM).

**Patients and methods:**

Ninety-three patients with newly diagnosed multiple myeloma and 23 controls had undergone routine magnetic resonance imaging (MRI) and diffusion-weighted MRI (DWI) from January 2019 to November 2020. Five bone marrow (BM) infiltration patterns were allocated according to routine MRI. The laboratory data and ADC values of patterns were analyzed and compared. ROC analysis was used to establish the best diagnostic ADC threshold value for identifying these patterns and distinguishing normal pattern from controls. Besides, the correlation between the ADC values of diffuse pattern and the plasma cells ratio was assessed.

**Results:**

The values of hemoglobin, beta-2 microglobulin (β2-MG), plasma cell, M protein, the percentages of stage, high-risk fluorescence in situ hybridization, and ADC values showed significant difference among patterns. ADC_mean_ at a specific value (368.5×10^−6^ mm^2^/s) yielded a maximum specificity (95.5%) and sensitivity (92.0%) in diagnosing MM. A specific value (335.5×10^−6^mm^2^/s) yielded a maximum specificity (84.7%) and sensitivity (88.0%) in discriminating visually normal pattern in MM from controls. There was a moderate positive correlation between the plasma cells ratio and ADCs of diffuse infiltration patterns (r = 0.648, P < 0.001).

**Conclusions:**

The bone marrow infiltration patterns in MM patients can indicate the tumor burden and ADC value has the ability to discriminate these patterns objectively.

## Introduction

Multiple myeloma (MM) is a malignant hematologic disease, and incidence is increasing gradually in recent years.^1^ It originates from B cells (plasma cells) and mainly invades bone marrow (BM).^2^ Tumor burden is important for treatment regimen and prognosis in MM patients. In clinical practice, tumor burden is assessed mainly by serum markers and BM biopsy.^3,4,5^ With the exposure of laboratory examination defects and the rapid development of imaging techniques, visual evaluation of BM infiltration degree and pattern becomes possible using imaging methods directly. As one of the most sensitive imaging methods, magnetic resonance imaging (MRI) can provide relevant information of BM infiltration.^6^ Whole body (WB) MRI has been listed as the golden standard for the detection of MM lesions by International Myeloma Working Group (IMWG), and many authorities around the world also supports its application in clinical practice for MM assessment and management.^7,8,9^

The latest IMWG guidelines divide the BM infiltration into five patterns based on MRI findings: focal involvement (F), pure diffuse infiltration (D), “salt-and-pepper” pattern (SP), combined diffuse/focal infiltration (M) and visually normal pattern (N).^10^ The pattern of BM infiltration at initial diagnosis could indicate the patient's tumor burden, which has important prognostic significance for MM patients.^11^ However, BM infiltration pattern recognition is a subjective assessment based on visual criteria. For example, D pattern is recognized by comparing with the signal intensity (SI) of the intervertebral disc or paravertebral muscle, and N pattern is not visually distinguishable from healthy BM.^12,13^ The lack of objective criteria may affect the interpretation of results and lead to false positive/negative diagnosis. With the appearance of diffusion-weighted MRI (DWI), it shows further sensitivity to detect MM lesions, and quantitative analysis of apparent diffusion coefficient (ADC) value becomes the hot spot for current research.^14,15^ However, the quantitative standards of ADC values of five patterns is lacking, especially for N pattern.

Furthermore, the IMWG criteria only considers the occurrence of F lesions as the beginning of clinical treatment, while the tumor burden and therapeutic value of other patterns, which includes D, SP, M, N, are still unclear. Our previous study found that the proportion of non-F patterns was significantly higher than that of F pattern.^16^ Therefore, the purpose of our study was to comprehensively explore and compare the tumor burden of different BM infiltration patterns. Furthermore, to evaluate the feasibility of ADC value in identifying these patterns in MM quantitatively.

## Subjects and methods

### Subjects

This study was approved by the Ethics Committee of the local institution (registration number: 000/2021). The newly diagnosed MM patients admitted to our institute from January 2019 to November 2020 were collected retrospectively. Inclusion criteria: (1) patients with MM confirmed by BM biopsy, immunofixation electrophoresis, serum protein electrophoresis and other laboratory examinations; (2) the patient underwent diffusion-weighted whole body MRI (WB-DWI) within one week before treatment; (3) Complete histological and laboratory data. Exclusion criteria: (1) patients with a history of additional malignant tumors; (2) patients who have received radiation therapy; (3) patients who have received granulocyte colony-stimulating factors or bisphosphonates; (4) patients with compressed fractures of vertebral body. At the same time, healthy controls were included. Inclusion criteria: (1) no history of malignant tumors; (2) no evidence of anemia in clinical and laboratory examinations; (3) age between 50 and 80.

### Histological and laboratory data

Marrow plasma cell ratio (%), M protein (g/L), β2-microglobulin (mg/L), hemoglobin (g/L), lactate dehydrogenase (LDH) (U/L), creatinine (μmol/L) levels, International Staging System (ISS) and Revised International Staging System (R-ISS) stage were collected. Fluorescence *in situ* hybridization (FISH) analysis after CDl38 separation, including Del(17p), t(4; 14), t(14; 16), t(14;20) and gain(1p) was also reported. MM patients were divided into high-risk cytogenetics (HRC) and standard-risk cytogenetics (SRC) groups on the basis of the FISH results.

### MR scanning protocol

All examinations were performed on a 3.0T MRI scanner (Magnetic Verio, Siemens Healthcare, Erlangen, Germany). Patients were in supine position with head first and arms placed on both sides of the body. The scanning parameters were as follows: coronal T2 TIRM sequence, repetition time: 7110 ms; echo time: 84 ms; slice thickness: 5 mm; slice spacing: 1.5 mm; FOV: 480 mm. The scan covered the skull, whole spine, pelvis and upper femur. DWI sequence, b values are 50 s/mm^2^ and 700 s/mm^2^, respectively. The scan range was the same as above. Sagittal T1 FSE sequence, repetition time: 1700 ms; echo time: 8.6 ms; slice thickness: 4 mm; slice spacing: 0.8 mm. Sagittal T2 FS sequence, repetition time: 3000 ms; echo time: 91 ms; slice thickness: 4 mm; slice spacing: 0.8 mm. The scan range was T11-S1. All DWI data was transferred to Syngo MultiModality station, and the Funtool software was used to process and generate ADC images.

### Image analysis

Images were analyzed by two radiologists with more than 10 years of experience. They were blinded to the laboratory data. Assessment differences between two radiologists were resolved by consensus.

**FIGURE 1.**
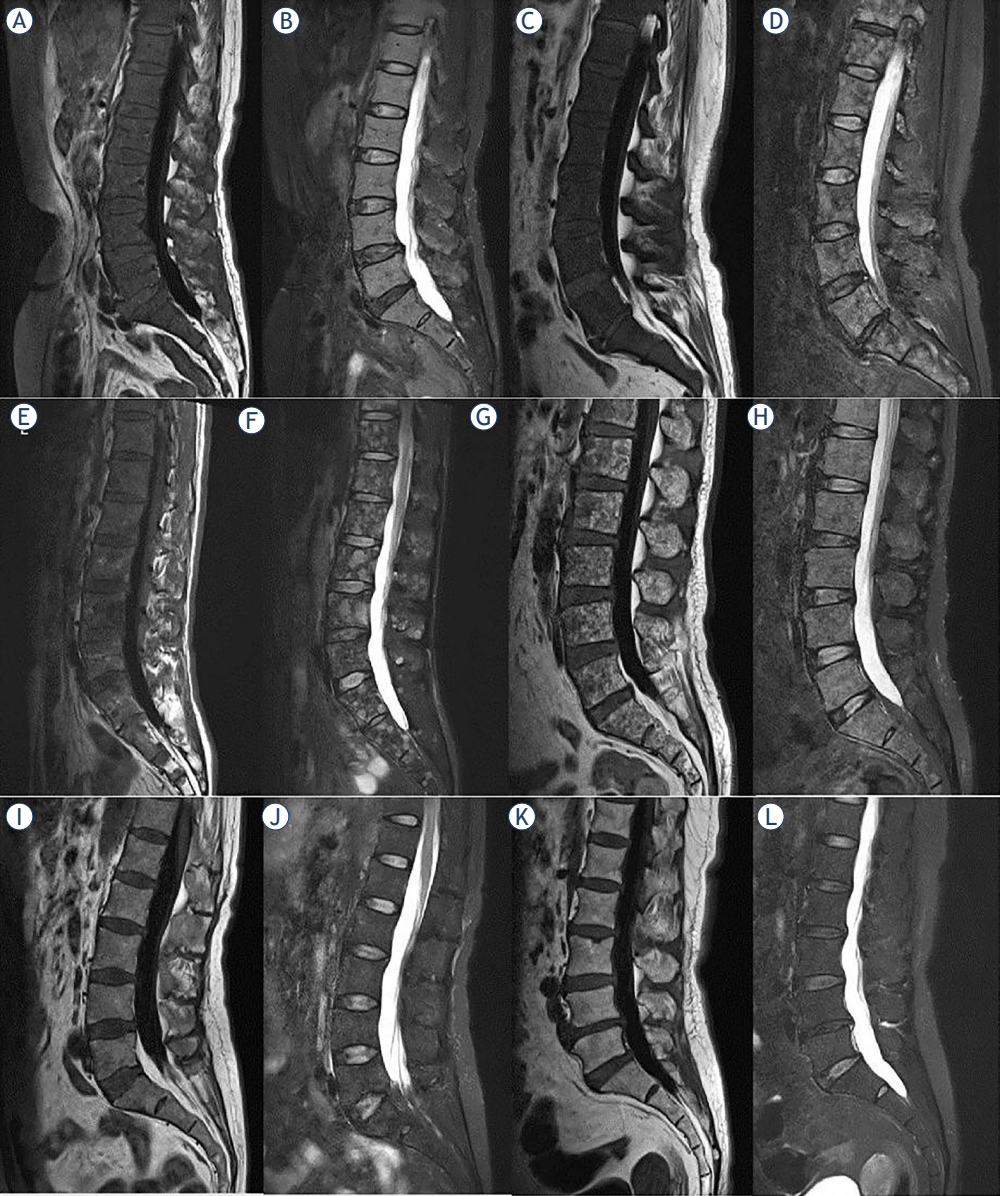
Five bone marrow infiltration patterns of multiple myeloma patients and healthy controls: pure diffuse pattern **(A, B)**, combined diffuse/focal pattern **(C, D)**, focal pattern **(E, F)**, salt and pepper pattern **(G, H)**, normal pattern **(I, J)**, healthy control **(K, L)**. T1 weighted(WI) image **(A, C, E, G, I, K)**, T2WI FS image **(B, D, F, H, J, L)**.

According to MRI findings, BM infiltration was divided into five patterns, including focal pattern (F), pure diffuse pattern (D), combined diffuse/focal pattern (M), salt and pepper pattern (SP) and normal pattern (N) (Figure 1). The detailed definition of patterns are as follows: F pattern was defined as signal intensity (SI) of nodular lesion less than or equal to the disc or surrounding muscles SI on T1WI and higher than that on T2 turbo inversion recovery magnitude (TIRM) images with diameter > 5mm. DWI (b = 700 s/mm^2^) images showed higher SI compared to peripheral BM. SP pattern was defined as sparse small foci with T1WI hypointense and T2 TIRM hyperintense with the background of normal vertebral body SI. D pattern was defined as the vertebral SI on the TIWI image less than or equal to the undegenerated disc or surrounding muscles SI without normal fat signal visible; on the T2 TIRM image, it shows diffusely higher SI. The M pattern was defined as the appearance of D pattern on the T1WI image and on the T2 TIRM image as one or more nodular higher SI within the vertebral body, where focal and diffuse changes were superimposed. The N pattern was defined as no visible SI change on vertebral T1WI and T2 TIRM images, which could not be distinguished from healthy BM by conventional MRI.

Since lumbar vertebra are one of the main sites for monoclonal plasma cell infiltration and characterized by lower amount of red marrow and more adipogenesis due to obvious mechanical stress and local ischemia, so we have chosen lumbar vertebra as representative background BM region. For MM patients with D, SP and M patterns, an oval ROI with 100 mm^2^ was placed in each vertebral body at the central slice in the ADC image with the aid of DWI (b values, 700) in conjunction with the anatomic (T1WI and T2WI FS) images by using image linking and scrolling workstation facilities and co-registration tools. Only when lesions detectable in these images, we will conduct ADC measurements. For MM patients with N pattern and healthy controls, we only need to place ROI with 100 mm^2^ in each vertebral body at the central slice in the ADC image since there is no abnormal SI detectable in these images. For F pattern, the lesions with clear boundaries and diameter of at least 5mm in the ADC image which also confirmed by other images were selected. ROI was placed on the largest slice of the lesion and contoured around the lesions as far as possible. Vertebral edge and BM edema, Schmorl's nodules and hemangioma were strictly avoided. ADC value of each ROI was measured three times at the same slice, and the average value of the three measurements was calculated and recorded.

### Statistical analysis

Statistical analysis was performed using IBM SPSS 25.0 software (Chicago, USA) and GraphPad Prism 5.0 software (GraphPad Software, California, USA). The normality of the distribution was assessed using the Kolmogorov-smirnov test. Oneway analysis of variance and Kruskal-Wallis H test were used to evaluate the differences between laboratory data of patients with different infiltration patterns. Chi-square test and Fisher's exact test were used to evaluate the difference of ISS stage, R-ISS stage and HRC status. Kruskal-Wallis H test was used to evaluate the difference of the mean ADCs between the control group and the MM group, and between different infiltration patterns. Pairwise comparison between multiple groups was performed by Bonferroni correction. Spearman correlation analysis was used to explore the correlation between ADC values of N, D patterns and the plasma cells ratio in BM. A P-value less than 0.05 was considered significant.

ROC curve was plotted to determine the cut-off values of ADC between healthy BM and MM BM, healthy BM and N pattern BM, N pattern BM and D pattern BM based on different research main concerns.

## Result

### Patients

Ninety-three MM patients were enrolled in this study and the laboratory data of these patients were collected (Table 1). The average age was 56.2 years and ranged from 31 to 85 years. Among them, 45 were males with an average age of 56.6 years, ranging from 34 to 85 years. There were 48 females with a mean age of 55.8 years, ranging from 31 to 79 years. At the same time, 23 healthy controls were included in the study with an average age of 59.6 years, ranging from 50 to 73 years. Among them, 11 were males with an average age of 59.1 years, ranging from 50 to 69 years. There were 12 female patients with a mean age of 60.0 years, ranging from 51 to 73 years. Among all patients, 23 were D pattern, 19 were M pattern, 17 were F pattern, 14 were SP pattern and 20 were N pattern.

**TABLE 1.** The laboratory data of patients

**Characteristics**	**Number / mean ± standard deviation**
**MM subtype^a^**	
lgG-K	28
lgG-λ	21
IgA-K	8
lgA-λ	11
IgM-K	1
K	5
λ	19
**ISS stage^a^**	
I/ II/ III	18/ 48/ 27
**R-ISS stage^a^**	
I/ II/ III	14/ 63/ 16
**FISH^a^**	
HRC/SRC	20 / 73
**Plasma cells in BM (%)^b^**	34.4 ± 22.3
**Serum M protein^b^**	36.6 ± 23.7
**Hemoglobin^b^**	93.3 ± 23.1
**Serum β2-MG^b^**	6.6 ± 8.5
**Serum creatinine^b^**	127.3 ± 143.3
**LDH^b^**	197.9 ± 115.1

a categorical variables shown with number;

b continuous variables shown with mean ± standard deviation

β2-MG = *beta-2* microglobulin; BM = bone marrow; FISH = fluorescence *in situ* hybridization; HRC = high-risk cytogenetics; ISS = international staging system; LDH = lactate dehydrogenase; MM = multiple myeloma; R-ISS = revised international staging system; SRC = standard-risk cytogenetics

### Clinical variables difference of patients with different infiltration patterns based on MRI

The relevant clinical variables of patients with different infiltration patterns were as follows: hemoglobin level was 93.3 ± 23.1 g/L, and the comparison of hemoglobin level among different infiltration patterns showed significant difference (Table 2). A total of 57 patients showed anemia clinically and the hemoglobin level decreased successively with the order of N, F, SP, M and D patterns. The creatinine level was 127.3 ± 143.3 g/L. There was no significant difference in serum creatinine level among different infiltration patterns, but the creatinine level still increased successively in SP, N, F, D and M patterns. The beta-2 microglobulin (β2-MG) level was 6.6 ± 8.5 mg/L. The comparison results of β2-MG level among patients with different infiltration patterns showed significant difference (Table 3). The β2-MG level increased successively according to the N, SP, F, D and M patterns. The plasma cells ratio in BM was 34.4 ± 22.3%. The comparison results among patients showed significant difference (Table 4). The plasma cells ratio in BM increased according to N, SP, F, D and M patterns. The M protein level was 36.6 ± 23.7 g/L. The comparison results among patients showed significant difference (Table 5). Serum M protein level increased according to N, F, SP, D and M patterns. The LDH level was 197.9 ± 115.1 U/L. There was no significant difference in LDH level among different infiltration patterns, but it still increased successively in N, SP, F, D and M patterns.

**TABLE 2.** Hemoglobin characteristics of multiple myeloma (MM) patients with different magnetic resonance imaging (MRI) infiltration patterns

**MRI pattern**	**Haemoglobin^a^ (g/L)**	***P***				

		**D**	**M**	**F**	**SP**	**N**
**D**	77.0 ± 19.5		> 0.05	0.001	0.014	< 0.001
**M**	88.9 ± 21.1			> 0.05	> 0.05	0.009
**F**	100.8 ± 17.8				> 0.05	> 0.05
**SP**	97.6 ± 20.0					> 0.05
**N**	106.7 ± 23.8					

a continuous variables shown with mean ± standard deviation

D = pure diffuse pattern; F = focal pattern; M = combined diffuse/focal pattern; N = normal pattern; SP = salt and pepper pattern

**TABLE 3.** *Beta-2* microglobulin (β2-MG) characteristics of multiple myeloma (MM) patients with different magnetic resonance imaging (MRI) infiltration patterns

**MRI pattern**	**β2-MG^a^ (mg/L)**	***P***				

		**D**	**M**	**F**	**SP**	**N**
**D**	9.3 ± 8.8		> 0.05	< 0.05	< 0.05	< 0.05
**M**	10.7 ± 13.9			< 0.05	< 0.05	< 0.05
**F**	4.4 ± 5.2				> 0.05	> 0.05
**SP**	2.9 ± 1.5					> 0.05
**N**	3.6 ± 2.4					

D = pure diffuse pattern; F = focal pattern; M = combined diffuse/focal pattern; N = normal pattern; SP = salt and pepper pattern

**TABLE 4.** Plasma cells characteristics of multiple myeloma (MM) patients with different magnetic resonance imaging (MRI) infiltration patterns

**MRI pattern**	**plasma cells^a^ (%)**	***P***				

		**D**	**M**	**F**	**SP**	**N**
**D**	46.9 ± 20.1		> 0.05	> 0.05	< 0.05	< 0.001
**M**	50.3 ± 23.1			< 0.05	< 0.05	< 0.001
**F**	34.9 ± 17.8				> 0.05	< 0.05
**SP**	18.8 ± 8.3					< 0.05
**N**	15.7 ± 11.2					

a continuous variables shown with mean ± standard deviation

D = pure diffuse pattern; F = focal pattern; M = combined diffuse/focal pattern; N = normal pattern; SP = salt and pepper pattern

**TABLE 5.** Serum M protein characteristics of multiple myeloma (MM) patients with different magnetic resonance imaging (MRI) infiltration patterns

**MRI pattern**	**M protein^a^ (g/L)**	***P***				

		**D**	**M**	**F**	**SP**	**N**
**D**	46.9 ± 27.6		> 0.05	> 0.05	> 0.05	< 0.05
**M**	49.8 ± 18.9			> 0.05	> 0.05	> 0.05
**F**	33.4 ± 20.8				> 0.05	> 0.05
**SP**	40.3 ± 18.8					< 0.05
**N**	19.9 ± 16.9					

a continuous variables shown with mean ± standard deviation

D = pure diffuse pattern; F = focal pattern; M = combined diffuse/focal pattern; N = normal pattern; SP = salt and pepper pattern

### ISS, R-ISS stage and HRC characteristics of MM patients with different infiltration patterns based on MRI

There were significant differences in ISS stage between groups (supplement Table 1). D, M and F patterns were mainly located on stage II and stage III, accounting for 91.3%, 94.7% and 82.4%, respectively. While N and SP patterns were mainly located on stage I and stage II, accounting for 85.0% and 92.9%, respectively.

There were significant differences in R-ISS stage between groups (Supplement Table 1). D and M patterns were mainly located on stage II and III, accounting for 100% and 94.7%, respectively. While F, N and SP patterns were mainly located on stage I and II, accounting for 94.1%, 90.0% and 100%, respectively.

A total of 20 patients with HRC status were detected with positive rate of 21.5%. The positive rate of HRC was 36.8% in M group, 30.4% in D group, 23.5% in F group and 10.0% in N group, respectively and no HRC cases were found in SP group. There were significant differences between groups and the detailed information of each patient with HRC status was collected (Supplement Table 2).

### ADC characteristics of MM patients with different infiltration patterns and controls based on MRI

A total of 440 ROIs were collected, of which 113 ROIs were collected from 23 patients with D pattern, 76 ROIs from 17 patients with F pattern, 100 ROIs from 20 patients with N pattern, 83 ROIs from 19 patients with M pattern and 68 ROIs from 14 patients with SP pattern. 111 ROIs were collected from 23 healthy controls.

The mean ADC value of the ROIs in MM patients was (631.4 ± 240.1)×10^−6^ mm^2^/s. Among them, the mean ADC values of D pattern were (607.8 ± 73.1) ×10^−6^ mm^2^/s, M pattern were (783.9 ± 196.4) ×10^−6^ mm^2^/s, F pattern were (967.8 ± 185.3) ×10^−6^ mm^2^/s, SP pattern were (463.9 ± 59.1) ×10^−6^ mm^2^/s, N pattern were (389.9 ± 63.8) ×10^−6^ mm^2^/s. The mean ADC of control group was (268.5 ± 63.6) ×10^−6^ mm^2^/s. Pairwise comparison of ADC values for different infiltration patterns and controls showed significant differences between groups.

### Diagnostic efficacy of quantitative ADC on distinguishing healthy BM and different BM infiltration patterns of MM patients

**FIGURE 2.**
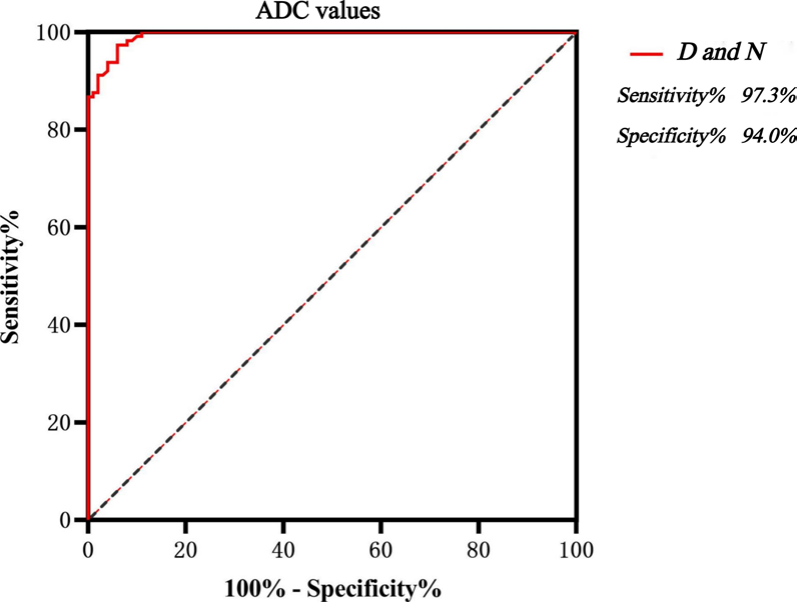
ROC curve analysis of discriminating multiple myeloma (MM) infiltration patterns of D from N by quantitative parameters of apparent diffusion coefficient (ADC). D = pure diffuse pattern; N = normal pattern

**FIGURE 3.**
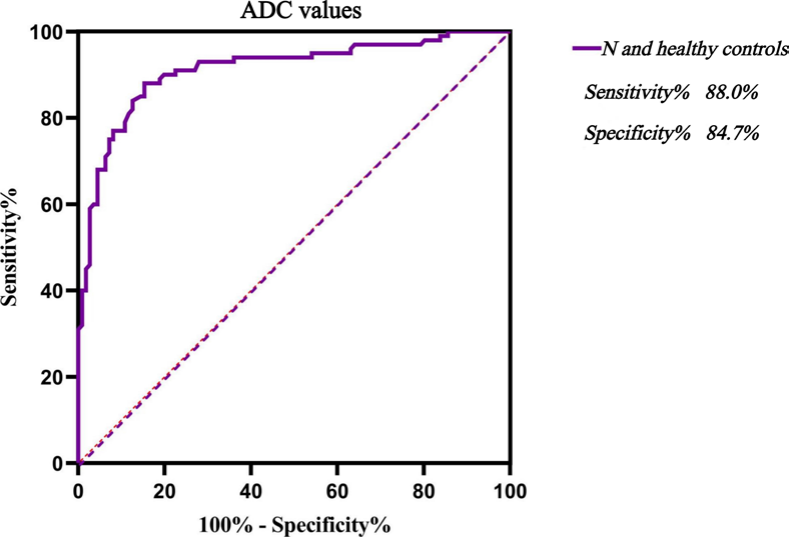
ROC curve analysis of discriminating multiple myeloma (MM) infiltration patterns of N from healthy controls by quantitative parameters of apparent diffusion coefficient (ADC). N = normal

**FIGURE 4.**
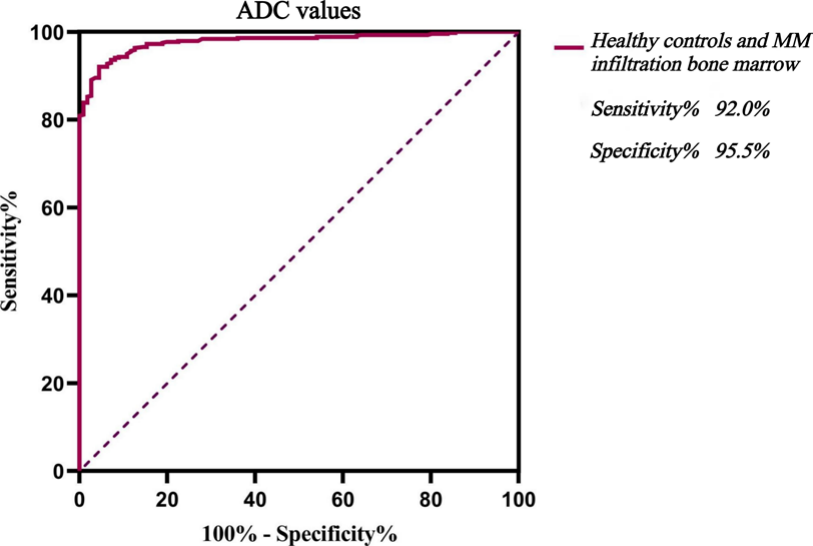
ROC curve analysis of discriminating healthy controls from multiple myeloma (MM) infiltration bone marrow by quantitative parameters of apparent diffusion coefficient (ADC).

An ADC value of 480.5 × 10^−6^ mm^2^/s was the optimal cut-off value for distinguishing D pattern from N pattern. The corresponding sensitivity was 97.3%, specificity 94.0%, and the area under the curve (AUC) was 0.994 (Figure 2). When the ADC value ≥ 522.5×10^−6^ mm^2^/s, the specificity of diagnosing D pattern was 100%. An ADC value of 335.5×10^−6^ mm^2^/s was the optimal cut-off value for distinguishing healthy BM from N pattern. The sensitivity was 88.0%, specificity 84.7%, and the AUC was 0.912 (Figure 3). When the ADC value ≥ 421.0×10^−6^ mm^2^/s, the specificity of diagnosing N pattern was 100%. An ADC value of 368.5×10^−6^ mm^2^/s was the optimal cut-off value for distinguishing healthy BM from MM BM, with diagnostic sensitivity of 92.0%, specificity of 95.5%, and AUC of 0.979 (Figure 4). When the ADC value ≥ 420.0 × 10^−6^ mm^2^/s, the specificity of diagnosing MM BM was 100%.

### Correlation between ADC value of D, N patterns and plasma cell ratio in BM

**FIGURE 5.**
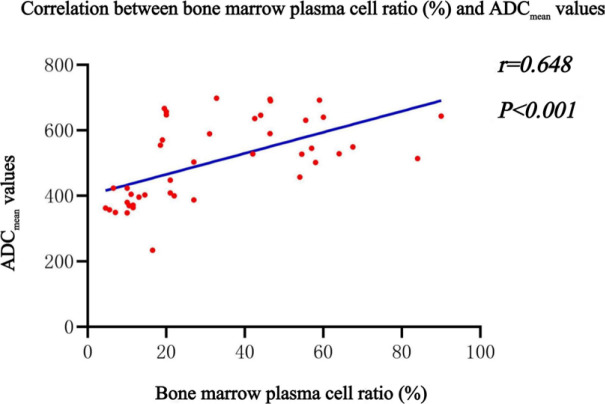
Correlation analysis of bone marrow plasma cell ratio and mean apparent diffusion coefficient (ADC_mean_) values of diffuse infiltration patterns (N and D) in multiple myeloma (MM) patients. D = pure diffuse pattern; N = normal pattern

ADC value of D, N patterns and plasma cell ratio in MM patients showed a moderate correlation with r = 0.648 (significance level P < 0.001) which indicates a positive correlation between plasma cell ratio and ADC value (Figure 5).

## Discussion

In this study, we aimed to explore the tumor burden of different patterns in MM patients and the ability of quantitative ADC values for discriminating different infiltration patterns and healthy BM. It showed that five different patterns in MM patients had different degrees of tumor burden, and it could be identified by quantitative analysis of ADC values.

Some studies have pointed out that the occurrence of MM is closely related to the BM microenvironment, and the serum tumor markers such as hemoglobin, β2-MG, creatinine could contribute to early clinical diagnosis and response evaluation.^17^ Anemia is one of the most common clinical presentations in patients and hemoglobin is used to evaluate anemia clinically.^18^ Renal function impairment is also one of the common clinical complications of MM. β2-MG and creatinine level have important implications in renal function impairment, and can also reflect the tumor burden of MM patients.^19,20,21^ Our results showed that D and M patterns had the highest β2-MG and creatinine levels and the lowest hemoglobin, which suggested that MM bone marrows of D or M patterns had more severe tumor burden than N and SP. The latest R-ISS stage incorporates cytogenetic abnormalities and elevated serum LDH based on the ISS stage.^22^ LDH can reflect the proliferative activity of tumors, which has been widely used in hematological malignancies such as MM, non-Hodgkin lymphoma and leukemia.^23,24^ Colovi and Perez-Andres found that LDH level is an independent prognostic indicator of MM through multivariate analysis.^25,26^ Our studies showed that the ISS and R-ISS stages of D and M patterns were significantly higher than the other three, While the other three types have no significant difference. It means that the prognosis of D and M patterns is worse than that of N, SP and F patterns. Moulopoulos *et al.* found that the survival period of D pattern was significantly shorter than those with F and N patterns.^5^ Tian *et al.* also demonstrated that D pattern had a worse prognosis than those with F pattern.^27^ These findings were consistent with our analysis, which may be related to the increased BM neoangiogenesis and advanced stage in MM patients with D pattern.

Despite the important prognostic significance of infiltration pattern for MM patients, it is still a subjective assessment based on the visual standard of disc or paravertebral muscle SI as a reference. The lack of objective criteria may affect the interpretation of the diagnostic results, especially for N pattern, which could not be distinguished from healthy BM by conventional MRI. With the development of DWI techniques, quantitative analysis of infiltrated BM is possible by measurement of ADC values. The results of this study showed that the healthy BM had the lowest ADC value. N pattern, unable to be visually recognized, had significantly higher ADC than that of healthy BM. The ADC value of MM BM increased sequentially based on N, SP, D, M, and F patterns. It is well known that normal fatty BM has low ADC value due to low water content and impeded water movement. In MM patients, increased ADC values are found which are characterized by reduced number of adipocytes, increased water content, and increased proportion of plasma cells.^15^ Thus, the more tumor cells infiltrate the vertebral body, the higher the ADC value and the tumor burden are.

D pattern was divided into different grades according to its severity by some scholars, including low grade, medium grade, high grade.^28^ The medium to high grade is defined as the lower SI of vertebral bone compared to the intervertebral disc on TlWI image. However, low-level infiltration has similar SI to healthy BM and is difficult to distinguish visually. Histological studies found that for diffuse infiltrating BM, when the plasma cells ratio was >50%, SI of vertebral body in TlWI was lower than the intervertebral disc. When plasma cells ratio is between 20% and 50%, the SI in TlWI image was slightly reduced and MM BM is unrecognizable visually from healthy BM.^29^ In our study, the plasma cells ratio of D pattern was 46.9 ± 20.1%, the vertebral SI was equal to or lower than that of the intervertebral disc in TlWI. The ratio of N pattern was 15.7 ± 11.23%, and the SI was higher than disc in T1WI. Since D and N patterns showed diffuse and homogenous performance in the conventional MRI images, we assumed that the examination of plasma cell ratio in BM could be replaced by use of ADC values for D and N patterns noninvasively. The results showed that the moderate correlation between ADC values of D, N patterns, and plasma cell ratio. Therefore, we believe that the N pattern defined in MRI corresponds to the lower level D pattern in histology, representing the initial stage of diffuse MM bone marrow infiltration. At this stage, BM plasma cell infiltration is mild, adipocytes is slightly reduced and the proportion of fat/water has little change, so the MM BM is indistinguishable visually. However, through the quantitative analysis of ADC value, it can be identified from the healthy BM, and its therapeutic value needs further exploration and research.

This study had several limitations. First, the scanning sequence does not include dynamic enhancement and in/out phase proposed in the literature because some MM patients with bone pain cannot bear long time scan and patients with renal insufficiency should not be injected intravenous contrast. Second, pathology cannot be performed on each lesion. Third, ADC cutoff values partly depend on the choice of b values of DWI images used for calculations. In our study, 2 b values (50 and 700) were used which may not reach an agreement in other institutions. Fourth, we have focused on the clinical variables which could reflect prognosis indirectly. Last, the observation range was limited to the lumbar spine since some studies found that it is the greatest important site of MM infiltration. In the future, we will expand the scope of the study and conduct a comprehensive and in-depth analysis in the prospective study with the aid of AI.

In summary, MM patients presenting with different BM infiltration patterns have different tumor burden and ADC values are able to identify infiltrated BM and further distinguish these patterns.

## Supplementary Material

Supplementary Material Details
