# Supplementary material for: Apparent diffusion coefficient measurements of bone marrow infiltration patterns in multiple myeloma for the assessment of tumor burden – a feasibility study
Source: Radiol Oncol. 2023 Nov 30;57(4):455–64. doi: 10.2478/raon-2023-0048 (PMC10690753; doi:10.2478/raon-2023-0048)
Supplement: Supplementary file 1 — Supplementary Material Details [file raon-2023-0048_sm.pdf]

sciendo Radiology and Oncology | Ljubljana | Slovenia | www.radioloncol.com
RADIOLOGY AND ONCOLOGY

Apparent diffusion coefficient measurements of bone marrow infiltration patterns in multiple myeloma for the assessment of tumor burden – a feasibility study

Xing Xiong, Yuzhu Ma, Yao Dai, Chunhong Hu, Yu Zhang

doi: 10.2478/raon-2023-0048

SUPPLEMENTARY TABLE 1. ISS and R-ISS stage characteristics of MM patients

Pattern[a]	D		M		F		SP		N	
	ISS	R-ISS	ISS	R-ISS	ISS	R-ISS	ISS	R-ISS	ISS	R-ISS
I	8.7%	0%	5.3%	5.3%	17.6%	17.6%	28.6%	21.4%	40.0%	35.0%
II	43.5%	69.6%	42.1%	63.2%	70.6%	76.5%	64.3%	78.6%	45.0%	55.0%
III	47.8%	30.4%	52.6%	31.6%	11.8%	5.9%	7.1%	0%	15.0%	10.0%

[a] categorical variables shown with number

D = pure diffuse pattern; F = focal pattern; ISS = international staging system; M = combined diffuse/focal pattern; MM = multiple myeloma; N = normal pattern; R-ISS = revised international staging system; SP = salt and pepper pattern;

SUPPLEMENTARY TABLE 2. The detailed information of MM patients with HRC status

No	Pattern	gain(1p)%	Del(13q14)%	Del(Rb1)%	Del(17p)	t(4; 14)	t(14; 16)	t(11; 14)
1	N	(-)	71	68	(-)	(+)	(-)	(-)
2	N	86	85	86	(-)	(+)	(-)	(-)
3	D	90	93	92	(-)	(+)	(-)	(-)
4	D	NA	NA	NA	(-)	(+)	(-)	(-)
5	D	92	92	92	(-)	(+)	(-)	(-)
6	D	(-)	42	45	(-)	(+)	(-)	(-)
7	D	(-)	(-)	(-)	(+)	(-)	(-)	(-)
8	D	(+)	(+)	(+)	(-)	(+)	(-)	(-)
9	D	(+)	(+)	(+)	(-)	(-)	(+)	(-)
10	F	(-)	90	90	(+)	(+)	(-)	(-)
11	F	(-)	82	83	(+)	(-)	(-)	(-)
12	F	82	80	82	(-)	(+)	(-)	(-)
13	F	80	82	80	(-)	(+)	(-)	(-)
14	M	87	92	89	(-)	(-)	(+)	(-)
15	M	(-)	(-)	(+)	(-)	(+)	(-)	(-)
16	M	22	20	22	(-)	(+)	(-)	(-)
17	M	(-)	(+)	(+)	(+)	(+)	(-)	(-)
18	M	(+)	(+)	(+)	(-)	(+)	(-)	(-)
19	M	(-)	72	75	(+)	(+)	(-)	(-)
20	M	(-)	90	92	(-)	(+)	(-)	(-)

% = the proportion of positive cells; + = positive; - = negative; NA = the data is missing

HRC = high-risk cytogenetic; MM = multiple myeloma
